# Characterization of the Oral and Stomach Microbial Community Structure in Patients with Intestinal Metaplasia, Dysplasia, and Gastric Cancer Through High-Throughput Sequencing

**DOI:** 10.3390/microorganisms14010209

**Published:** 2026-01-16

**Authors:** Hokyung Song, Seon Woo Oh, Jung-Hwan Oh, Tatsuya Unno

**Affiliations:** 1 Department of Environmental Engineering, Chosun University, Dong-gu, Gwangju 61452, Republic of Korea; hk.song15@chosun.ac.kr; 2 Institute of Well-Aging Medicare & CSU G-LAMP Project Group, Chosun University, Gwangju 61452, Republic of Korea; 3 Department of Internal Medicine, Eunpyeong St. Mary’s Hospital, College of Medicine, The Catholic University of Korea, Seoul 03312, Republic of Korea; strabie@naver.com; 4 Department of Biological Sciences and Biotechnology, Chungbuk National University, Seowon-gu, Cheongju 28644, Republic of Korea

**Keywords:** gastric cancer, stomach microbiome, oral microbiome, intestinal metaplasia, dysplasia, 16S rRNA gene, highthroughput sequencing

## Abstract

Gastric cancer (GC) is the fifth most common cancer worldwide, with the highest incidence in East Asia. Although *H. pylori* is a well-known risk factor, carcinogenesis can occur independently of *H. pylori* infection, and approximately 43% of adults carry *H. pylori* as part of their native microbiota. This study aimed to identify potential oral and gastric microbial markers across different histological stages of GC in both *H. pylori*-positive and -negative patients. Buccal swabs and gastric mucosa samples were collected from patients with intestinal metaplasia, low-grade dysplasia, high-grade dysplasia, early GC, or advanced GC. Total DNA was extracted, and 16S rRNA gene amplicon sequencing was performed. Microbiome diversity generally remained stable across histological stages, with no directional shifts in community structure. Differential abundance analysis revealed higher relative abundances of *Anaerostipes*, *Phocaeicola*, and *Collinsella* in the gastric antrum of cancerous samples. *Anaerostipes* and *Phocaeicola* are typically enriched in the intestinal microbiota but are rarely observed in the stomach, suggesting their potential ecological and pathological relevance in gastric carcinogenesis. In *H. pylori*-negative patients, however, a different stage-associated abundance pattern was observed, in which *Faecalibacterium*, a genus predominantly associated with the intestinal environment, was less abundant in advanced gastric cancer samples than in earlier histological stages within the gastric body. These findings suggest that microbial changes during gastric cancer progression may follow different trajectories depending on *H. pylori* infection status. In oral samples, *Haemophilus* and *Prevotella* were more abundant in intestinal metaplasia than in low-grade dysplasia, and network analysis indicated links between *Neisseria* and *Filifactor* at oral and gastric sites. However, as the study population was limited to a single country and ethnicity, the applicability of these microbial markers should be carefully considered.

## 1. Introduction

Gastric cancer remains a major global health burden, particularly in East Asian countries [1]. In current clinical practice, the eradication of *Helicobacter pylori* infection is a well-established strategy for preventing gastric cancer [2]. Numerous past studies have demonstrated that successful *H. pylori* eradication reduces the incidence of gastric malignancy by interrupting the chronic inflammatory cascade, leading to carcinogenesis [3,4,5].

However, *H. pylori* infection alone cannot fully explain the complex mechanisms underlying gastric carcinogenesis. A substantial proportion of gastric cancer cases occur despite prior *H. pylori* eradication or even in the absence of detectable infection, thus suggesting that other microbial- or host-related factors contribute to malignant transformation [4,6,7]. Therefore, identifying reliable biomarkers that can predict the risk of gastric cancer independent of *H. pylori* status has become a critical issue in clinical practice [7,8].

From a clinical perspective, biomarkers that can be detected through simple and non-invasive sampling methods, such as buccal swab collection, would markedly enhance the feasibility of large-scale screening and surveillance programs. In this context, increasing evidence has suggested that the oral microbiota may serve as a promising non-invasive biomarker for gastric diseases. For example, Liu et al. (2021) [9] reported distinct oral microbiome profiles between patients with functional dyspepsia and healthy controls, proposing Spirochaetes, *Kingella*, and *Abiotrophia* as potential diagnostic markers. Wu et al. (2022) [10], using shotgun metagenomic sequencing, identified oral taxa such as *Peptostreptococcus stomatis* and *Johnsonella ignava* that were enriched in patients with intestinal metaplasia. More recently, machine learning-based approaches have further highlighted the diagnostic potential of oral microbiota, with Oh et al. (2025) [11] developing a classifier capable of discriminating multiple gastrointestinal cancers based on oral microbial signatures, and Gao et al. (2025) [12] identifying oral bacterial markers associated with gastric cancer using a deep neural network model.

In parallel, accumulating evidence has emphasized the role of both gastric and oral microbiota in the pathogenesis of gastric cancer, suggesting that alterations in oral microbial communities may reflect changes in the gastric microenvironment [13,14,15,16]. Despite these advances, several important gaps remain in the current literature. Most existing studies have focused either on the oral or gastric microbiota in isolation, or have examined a single disease entity, such as intestinal metaplasia or gastric cancer, without systematically considering the stepwise histological progression of gastric carcinogenesis.

Therefore, this study aimed to comprehensively characterize the gastric mucosal microbiota in patients with intestinal metaplasia, gastric dysplasia, and gastric cancer by analyzing biopsy specimens obtained from both the antrum and body. In addition, we sought to explore the potential of buccal swab-derived oral microbiota as a non-invasive biomarker for predicting gastric carcinogenic progression through an integrated analysis of oral and gastric microbial profiles across distinct histological stages.

## 2. Materials and Methods

### 2.1. Study Population and Sample Collection

This study included 83 patients who underwent upper gastrointestinal endoscopy at Eunpyeong St. Mary’s Hospital in South Korea. The enrolled patients were categorized into three different diagnostic groups based on the histological confirmation of gastric biopsy specimens: intestinal metaplasia, gastric dysplasia, and gastric cancer. For detailed analysis, the patients were further subdivided into five groups: intestinal metaplasia (IM), low-grade dysplasia (LGD), high-grade dysplasia (HGD), early gastric cancer (EGC), and advanced gastric cancer (AGC).

Buccal swab samples were collected immediately prior to upper endoscopy after routine pre-endoscopic preparation, which included general oral hygiene measures such as tooth brushing but excluded dental procedures or antiseptic mouth rinses. Buccal swab sampling was performed by gently rubbing sterile swabs against the inner surfaces of both cheeks. During the procedure, gastric mucosal biopsy specimens were systematically obtained from two anatomical sites: one specimen from the lesser curvature side of the antrum and one from the greater curvature side of the mid-body. The samples were then promptly transferred to sterile tubes and stored at −80 °C for the subsequent analysis.

### 2.2. Endoscopic Assessment and Classification of Gastritis

Patients were instructed to discontinue antibiotics and proton pump inhibitors for at least one week prior to endoscopy. During endoscopy, the degree and pattern of gastritis were evaluated using the Kyoto classification. This system assesses five endoscopic features: atrophy, intestinal metaplasia, enlarged gastric folds, nodularity, and diffuse redness. The sum of these scores constituted the total Kyoto score, which ranged from 0 to 8. A higher Kyoto score reflects more severe gastritis and is associated with an increased risk of *H. pylori* infection and gastric cancer development [17].

### 2.3. Detection of Helicobacter pylori

The presence of *H. pylori* in gastric biopsy specimens was confirmed using either silver staining (Warthin-Starry staining) or PCR-based detection methods targeting specific *H. pylori* genes. A U-TOP HPy-ClaR detection kit (Seasun Biomaterials, Daejeon, Republic of Korea) was used for PCR-based detection. In some patients, *H. pylori* was not detected by silver staining or PCR; however, the genus *Helicobacter* showed a high relative abundance in 16S rRNA amplicon sequencing. Because species-level resolution is limited with Illumina MiSeq, *H. pylori* status in such cases was determined based on silver staining or PCR results.

### 2.4. DNA Extraction and 16S rRNA Gene Amplicon Sequencing

Sample DNAs were extracted using the QIAamp DNA Mini Kit (QIAGEN, Hilden, Germany). The sequencing library was prepared following the “16S Metagenomic Sequencing Library Preparation” protocol provided by Illumina (https://support.illumina.com/downloads/16s_metagenomic_sequencing_library_preparation.html, assessed on 1 March 2022). First, the V3–V4 region of the 16S rRNA gene was amplified using the 16S Amplicon PCR Forward Primer (5′-TCGTCGGCAGCGTCAGATGTGTATAAGAGACAGCCTACGGGNGGCWGCAG-3′) and Reverse Primer (5′-GTCTCGTGGGCTCGGAGATGTGTATAAGAGACAGGACTACHVGGGTATCTAATCC-3′). The amplicon PCR condition was as follows: (1) initial denaturation at 95 °C for 3 min, (2) 25 cycles of denaturation (95 °C, 30 s), annealing (55 °C, 30 s), and elongation (72 °C, 30 s), and (3) final elongation at 72 °C for 5 min. PCR products were purified using HiAccuBeads (AccuGene, Incheon, Republic of Korea). Index PCR was performed using the Nextera XT Index Kit V2 (Illumina, San Diego, CA, USA), followed by cleaning. The sample library was pooled and sequenced on an Illumina MiSeq platform. The samples with insufficient DNA content were excluded (Supplementary Table S1). Raw FASTQ files were deposited in the National Center for Biotechnology Information (NCBI) Sequence Read Archive (SRA) with the accession number PRJNA1380972.

### 2.5. Sequence Processing

Raw FASTQ read files were processed using the Mothur MiSeq SOP (https://mothur.org/wiki/miseq_sop/; accessed on 24 January 2024). Sequence reads longer than 550 bp or shorter than 350 bp were excluded from analysis. Sequence reads with ambiguous bases or homopolymers (eight base pairs) were removed. RDP database v. 2.13 [18] was used for taxonomic classification of each read, and sequences classified as “Eukaryota”, “Chloroplast”, “Mitochondria”, and “unknown” were removed. The operational taxonomic units (OTUs) were defined using a sequence similarity cutoff of 97%. The samples with insufficient sequence reads (<10,000) were excluded from further analyses (Supplementary Table S1).

### 2.6. Statistical Analysis

Phylum-level and genus-level community compositions were visualized as heatmaps using the ‘pheatmap’ package in R v4.5.1. Prior to calculating diversity of the microbial communities, the sequences were subsampled with 10,043 reads per sample. Analysis of variance (ANOVA) was performed in order to test for significant differences in alpha diversities and Firmicutes/Bacteroidetes (F/B) ratio among histological stages within each sampling point, and Tukey’s honestly significant difference (HSD) test was conducted as a post hoc test when the ANOVA result was found to be significant. Non-metric multidimensional scaling (nMDS) plots were generated based on the Bray–Curtis distance between samples, which was calculated from the square-root transformed OTU compositional data. Personal characteristics were plotted onto the nMDS ordination using the ‘envfit’ function in the ‘vegan’ package in R to identify associations between personal characteristics and microbial communities. Permutational multivariate analysis of variance (PERMANOVA; ADONIS) was conducted to determine whether there were any significant differences in the microbial communities between the sample groups. Pairwise ADONIS analysis was performed using the ‘pairwise.adonis’ function in the ‘pairwiseAdnois’ package in R when the PERMANOVA result was significant. Differential abundance analysis was performed between pairs of histological stages using the “DESeq” function in the R “DESeq2” package. A co-occurrence network was generated based on Spearman’s correlation between the relative abundances of genera at each sampling point. Only genera with relative abundances greater than 0.001 were included in the network analysis. The network was visualized using Cytoscape v3.10.2.

## 3. Results

**Figure 1:**
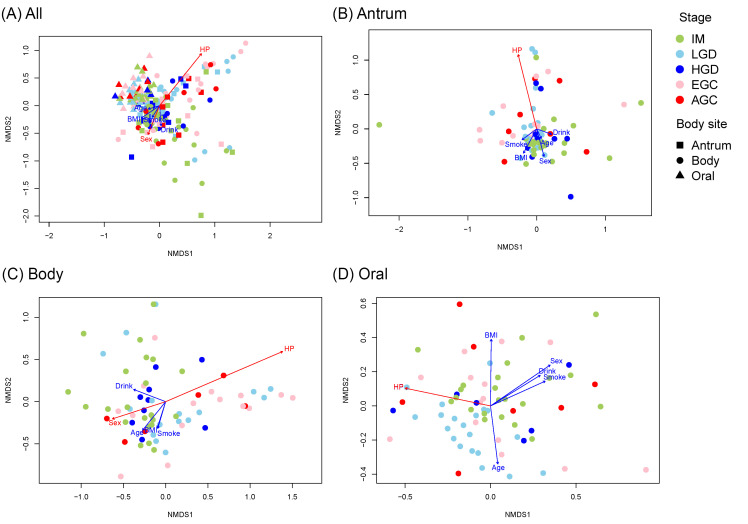
nMDS plots representing the Bray–Curtis distance between the samples, calculated based on the square-root transformed abundance of each OTU in each sample.

The nMDS and PERMANOVA results showed that the oral microbial communities were distinct from those in the antrum and body (Figure 1A and Table S2), whereas the antrum and body communities did not differ significantly. *H. pylori* status (presence vs. absence) and sex were found to be significantly associated with microbial community structure (Figure 1A and Table S3). In the antrum, no pairwise differences were observed between histological stages (Figure 1B and Table S4); however, *H. pylori* status was significantly associated with nMDS ordination (Figure 1B and Table S5). The microbial community structures differed significantly between the IM and EGC groups (Figure 1C and Table S6), and both *H. pylori* status and sex were significantly associated with nMDS ordination (Figure 1C and Table S7). In the oral samples, the IM and LGD differed significantly in the community structure (Figure 1D and Table S8), and *H. pylori* status again correlated with ordination (Figure 1D and Table S9).

**Figure 2:**
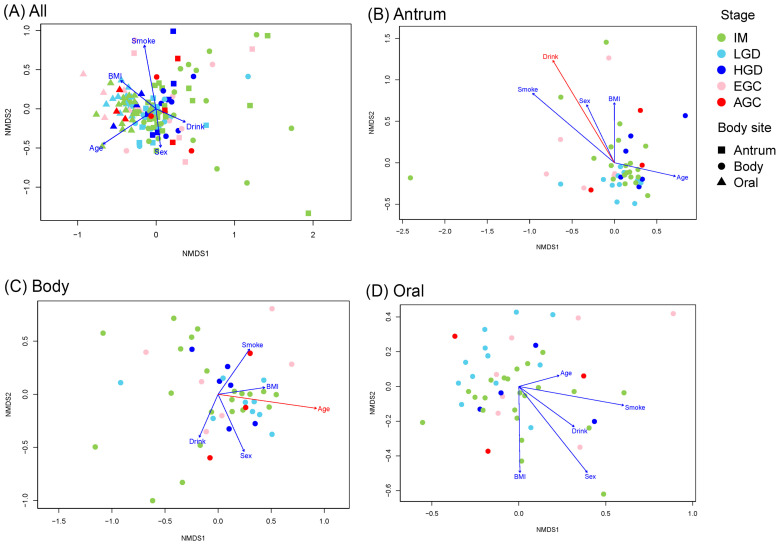
nMDS plots representing the Bray–Curtis distance between the *Helicobacter pylori*-negative samples, calculated based on the square-root transformed abundance of each OTU in each sample.

When analyzing only the *H. pylori*-negative samples, the oral communities remained distinct from the antrum and body (Figure 2A and Table S10), and no personal characteristics showed any significant correlations with ordination (Figure 2A and Table S11). In the antrum, microbial communities did not differ by stage (Figure 2B and Table S12), but alcohol consumption (“Drink”) was significantly associated with the ordination (Figure 2B and Table S13). Similarly, no stage-related differences were found in the body (Figure 2C and Table S14), but age was significantly correlated with ordination (Figure 2C and Table S15). In the oral samples, the IM and LGD differed significantly (Figure 2D and Table S16), but no personal characteristics were associated with ordination (Figure 2D and Table S17).

**Figure 3:**
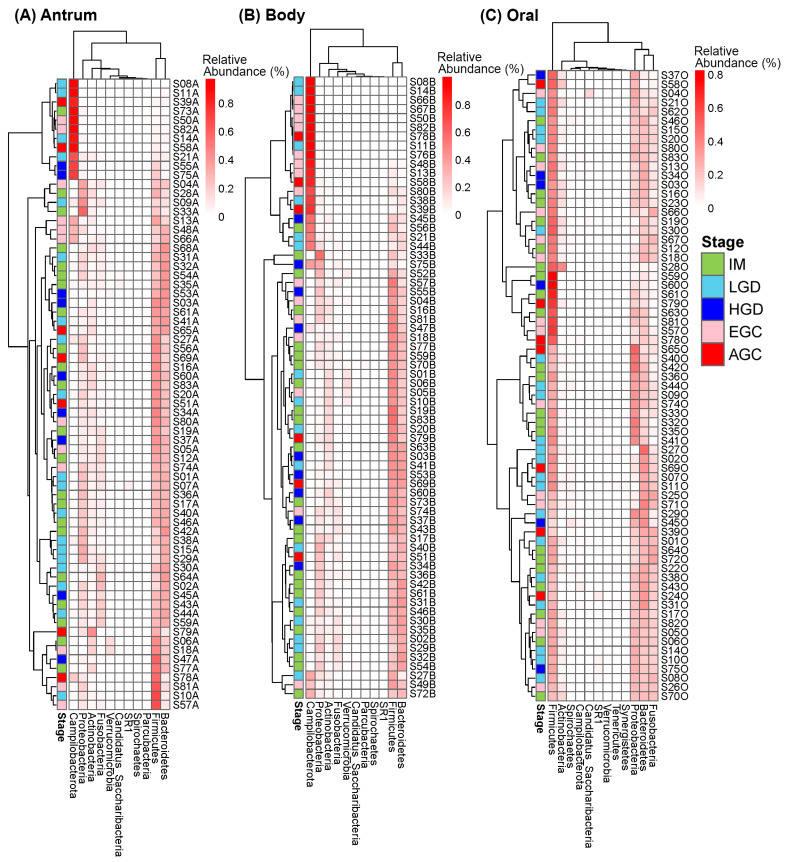
Heatmaps of the top 10 most abundant phyla in the (**A**) gastric antrum, (**B**) gastric body, and (**C**) oral samples, excluding taxa that could not be classified at the phylum level.

**Figure 4:**
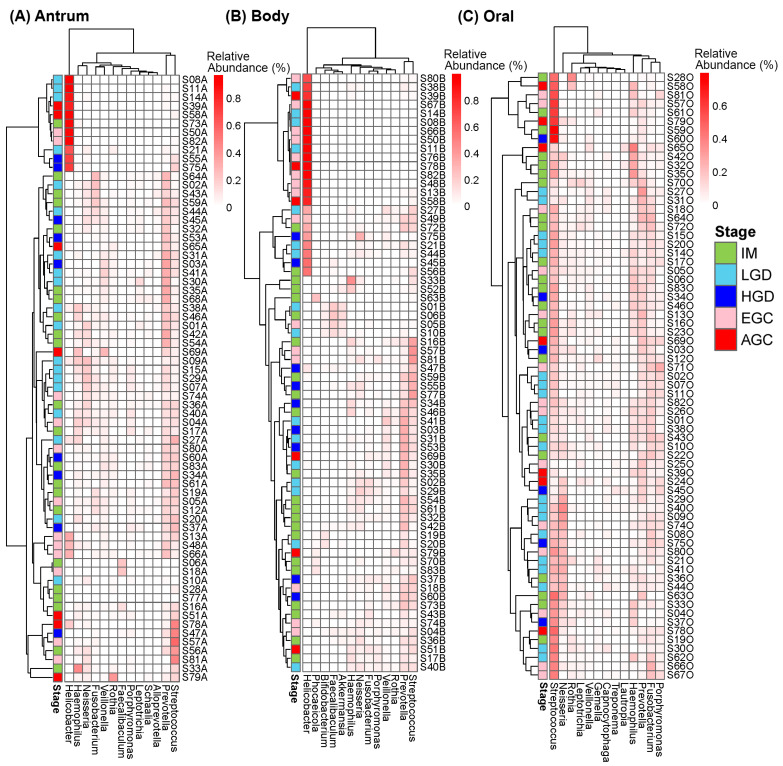
Heatmaps of the top 13 most abundant genera in the (**A**) gastric antrum, (**B**) gastric body, and (**C**) oral samples, excluding taxa that could not be classified at the genus level.

At the phylum level, the microbial communities in the antrum and body were dominated by Firmicutes, Bacteroidetes, Campilobacterota, and Proteobacteria (Figure 3A,B). In contrast, the oral microbiome was dominated by Firmicutes and Bacteroidetes, followed by Proteobacteria and Fusobacteria (Figure 3C). There were no significant differences seen in the Firmicutes/Bacteroidetes (F/B) ratios among the histological stages at each sampling site, except between the LGD and EGC in the body microbiome (Figure S1). At the genus level, microbial communities in the antrum and body were found to be dominated by *Helicobacter*, *Prevotella*, *Streptococcus*, *Haemophilus*, and *Neisseria* (Figure 4A,B). The oral microbiome was dominated by *Streptococcus*, *Prevotella*, *Fusobacterium*, *Haemophilus*, and *Neisseria* (Figure 4C). *Helicobacter* was only minimally found in oral samples with an average relative abundance of 0.00018 (±0.00084 SD).

**Figure 5:**
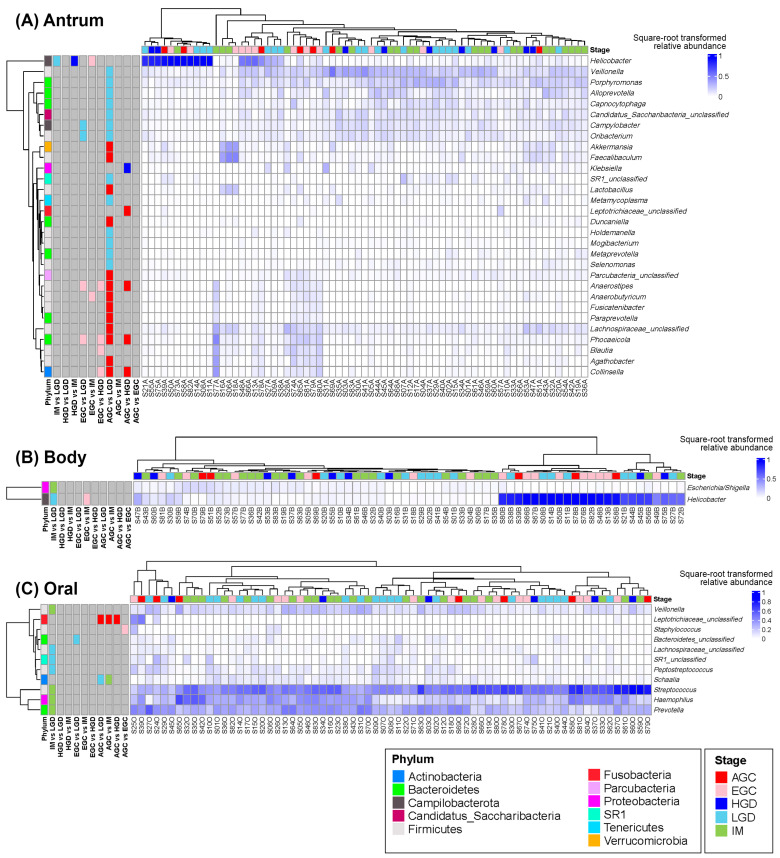
Heatmaps showing the square-root-transformed relative abundances of differentially abundant genera across histological stages in the (**A**) gastric antrum, (**B**) gastric body, and (**C**) oral samples. The leftmost annotation for each row indicates the phylum to which each genus belongs. The remaining row annotations represent the results of pairwise differential abundance analyses. Stages with higher abundance are color-coded, whereas stages with lower abundance are shown in gray.

The results of the differential abundance analysis revealed *Helicobacter* to be one of the most distinguishing genera across the histological stages in the antrum and body (Figure 5). In the antrum, *Helicobacter* was more abundant in the LGD, HGD, and EGC than in the IM, whereas in the body, it was more abundant in the LGD and EGC than in the IM. Other genera showing stage-specific differences in the antrum included *Anaerostipes*, *Phocaeicola*, and *Collinsella*, which were more abundant in the cancer samples (EGC or AGC) than in dysplastic samples (LGD or HGD). In the oral samples, *Streptococcus*, *Haemophilus*, *Prevotella*, and *Veillonella* were more abundant in IM than in the LGD, whereas *Peptostreptococcus* was more abundant in the LGD than in IM.

**Figure 6:**
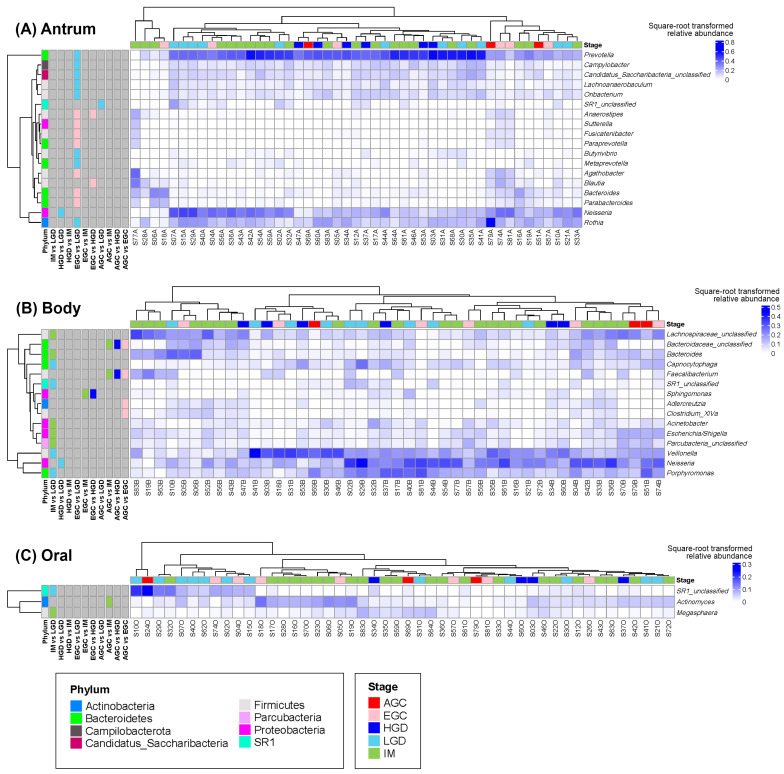
Heatmaps showing the square-root-transformed relative abundances of differentially abundant genera across histological stages in *Helicobacter pylori*-negative samples in the (**A**) gastric antrum, (**B**) gastric body, and (**C**) oral samples. The leftmost annotation for each row indicates the phylum to which each genus belongs. The remaining row annotations represent the results of pairwise differential abundance analyses. Stages with higher abundance are color-coded, whereas stages with lower abundance are shown in gray.

Among *H. pylori*-negative samples, significant differences were observed mostly in the comparison between early gastric cancer (EGC) and low-grade dysplasia (LGD) in the antrum (Figure 6). Genera such as *Prevotella*, *Campylobacter*, *Lachnoanaerobaculum*, *Oribacterium*, *Butyrivibrio*, *Metaprevotella*, and *Rothia* were more abundant in LGD, whereas *Anaerostipes*, *Sutterella*, *Fusicatenibacter*, *Paraprevotella*, *Agathobacter*, *Bacteroides*, and *Parabacteroides* were enriched in EGC. In the gastric body, *Faecalibacterium* was less abundant in AGC than in IM, HGD, and EGC. Compared with LGD, *Bacteroides*, *Acinetobacter*, and *Escherichia/Shigella* were more abundant in intestinal metaplasia (IM), while *Capnocytophaga*, *Veillonella*, and *Porphyromonas* were relatively more abundant in LGD. In oral samples, *Megasphaera* was more abundant in IM than in LGD, whereas *Actinomyces* was more abundant in IM than in AGC.

**Figure 7:**
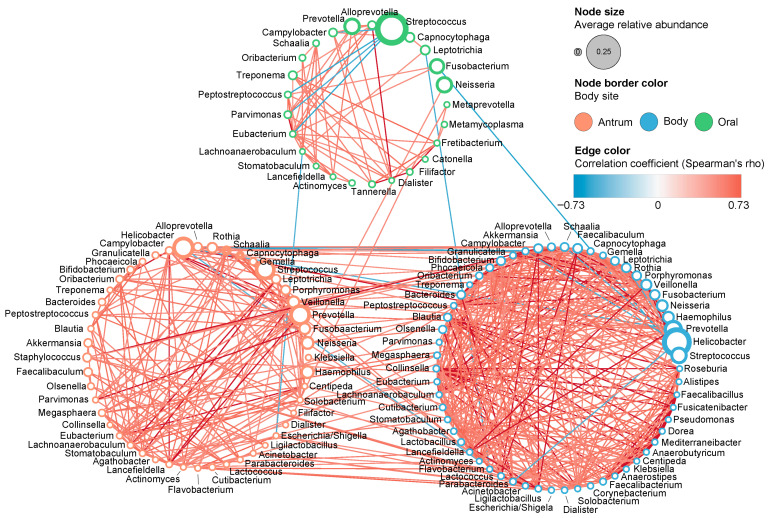
Co-occurrence networks of the microbial genera in oral, antrum, and body samples. Among the significant correlations (*p* < 0.05), only those with Spearman’s rho values greater than 0.5 or less than −0.5 are shown.

Co-occurrence network analysis revealed a strong association between the microbial genera of the antrum and body (Figure 7). Most correlations found between the antrum and body samples were positive, except for *Helicobacter*, which constituted over 50% of the antrum and body microbiome in some patients (Figure 4). Only a small number of genera showed correlations between the oral and antrum samples or between the oral and body samples. Significant positive correlations were found between *Neisseria* (oral) and *Neisseria* (antrum), and between *Filifactor* (oral) and *Filifactor* (antrum). Significant negative correlations were found between *Lachnoanaerobaculum* (oral) and *Escherichia*/*Shigella* (antrum), *Leptotrichia* (oral) and *Flavobacterium* (body), and *Fusobacterium* (oral) and *Streptococcus* (body).

The overall diversity of the microbial communities did not differ between the stages (Figures S2 and S3). Significant differences were observed only in the body, where the EGC samples showed a lower number of OTUs than IM and HGD and lower Shannon diversity than IM.

## 4. Discussion

Many previous studies have reported significant differences in microbial community composition and diversity with the progression of gastric cancer [19,20]. While some studies have observed a decrease in microbial diversity as cancer develops [21,22], our study showed stable diversity patterns across the developmental stages. In addition, in our study, both stomach and oral microbiomes showed no directional shifts in gastric cancer development, and the community-level pairwise differences were generally insignificant (Figure 1, Tables S4, S6 and S8). This pattern may be due to the high inter-individual variability influenced by personal history, lifestyle, dietary habits, or genetic background, which could mask microbial changes associated with gastric cancer progression. Nevertheless, the performed differential abundance analysis revealed several genera that varied significantly across the histological stages (Figure 5).

As previously reported, *Helicobacter* showed a strong association with the histological stage of gastric cancer development. In both the antrum and body, *Helicobacter* tended to be more abundant at the other stages than in the IM (Figure 5). Several studies have suggested that *H. pylori* produces ammonia via urease activity, which neutralizes gastric acid and contributes to changes in the gastric environment [23]. This can lead to gastric atrophy and intestinal metaplasia, followed by a shift toward intestinal-type microbial communities [24,25].

Other differentially abundant genera included *Anaerostipes*, *Phocaeicola*, and *Collinsella*, which were more abundant in the antrum of patients with gastric cancer (Figure 5). *Anaerostipes* and *Phocaeicola* are commonly found in the intestine, where they contribute to short-chain fatty acid (SCFA) production, which is generally considered beneficial for gut health [26,27,28,29]. However, their presence and role in the stomach are not well characterized. The enrichment of intestinal-associated taxa such as *Anaerostipes* and *Phocaeicola* in gastric compartments may reflect a progressively altered gastric environment characterized by reduced acidity and mucosal atrophy, conditions that are known to precede neoplastic transformation [30,31]. Such microbial shifts may therefore serve as indirect indicators of ongoing mucosal changes rather than direct oncogenic drivers. In contrast, *Collinsella* has often been described as a potentially harmful taxon, as it can increase gut permeability and alter cholesterol metabolism, and has been associated with type 2 diabetes and rheumatoid arthritis [32]. Our findings consistently indicate a potential association between *Collinsella* spp. and gastric cancer.

In the oral samples, *Streptococcus*, *Haemophilus*, *Prevotella*, and *Veillonella* were more abundant in IM when compared to LGD, whereas *Peptostreptococcus* was more abundant in the LGD, thus suggesting that these genera may serve as oral microbial markers distinguishing between the IM and LGD. However, oral microbial markers associated with gastric cancer progression are relatively underexplored, and further data are needed to validate their relevance. In a study by Huang et al. (2021) [33], patients with gastric cancer showed a decreased relative abundance of *Haemophilus*, *Prevotella*, *Peptostreptococcus*, and others in the oral microbiome compared to patients with superficial or atrophic gastritis. They suggested that *Haemophilus* and *Prevotella* may play protective roles through nitrate reduction, resulting in the accumulation of carcinogenic N-nitroso compounds. However, in our samples, these genera did not show clear enrichment in cancer cases. This implies that the roles of these taxa may depend on disease stage, and stage-specific analyses are necessary to clarify their association with gastric cancer development.

When the microbiomes of *H. pylori*-negative patients were compared, several individual characteristics were associated with differences in microbial community structure. For example, the antral microbiome correlated with alcohol consumption, whereas the body microbiome was associated with age (Figure 2, Tables S13 and S15). In the gastric body, *Faecalibacterium* was less abundant in AGC than in IM, HGD, and EGC. This finding contrasts with the results from analyses including all samples regardless of *H. pylori* status, in which *Anaerostipes* and *Phocaeicola* were more abundant in cancerous samples. Notably, all three genera are well-known SCFA-producing bacteria generally considered beneficial in the intestinal environment [27,28,29,34], yet they are uncommon in the stomach. Therefore, the opposite trends observed in *H. pylori*-negative conditions suggest that the gastric microbial context—and its association with carcinogenesis—may differ from that observed in the overall cohort, supporting the possibility that *H. pylori*-negative gastric carcinogenesis follows distinct microbial and pathogenic pathways. In the oral samples, *Megasphaera* was more abundant in IM than in LGD, and *Actinomyces* was more abundant in IM than in AGC, both of which have been associated with mucosal atrophy [20]. These taxa may be involved in the early stages of gastric lesion development (IM) but may not necessarily contribute to cancer progression.

The community structure of the oral microbiome was found to be clearly distinct from that of the antrum and body, and the network analysis suggested that oral–gut microbial linkage was not a major determinant of gastric microbiome composition. However, some signals indicated the possible transmission or shared dynamics of specific bacterial genera between the oral and gastric environments (Figure 7). For example, the relative abundances of *Neisseria* and *Filifactor* in the oral cavity positively correlated with those in the antrum. A positive association between *Neisseria* in the gut and precancerous gastric lesions has been reported [20]. However, in our samples, *Neisseria* was differentially abundant in LGD when compared to HGD in *H. pylori*-negative cases, and no other stage-related differences were found (Figure 6). Although direct comparisons are limited by the absence of healthy controls in our study, these observations suggest that *Neisseria* could be involved in the intermediate stages of precancerous progression, with a higher abundance in LGD that decreases as lesions advance to HGD.

Although fewer oral–gastric correlations were observed in the co-occurrence network analysis than anticipated, a low degree of correlation does not necessarily mean that the oral microbiome cannot serve as a proxy for the gastric conditions. Given the temporal and spatial dynamics of microbial exposure, oral microbes may transiently influence the gastric environment, potentially leading to downstream changes in gastric microbial composition or host responses. Therefore, limited oral–gastric co-occurrence alone may not be sufficient to rule out the potential relevance of oral taxa as disease-associated biomarkers.

Importantly, we identified several oral microbial signatures that were clearly stage-specific with respect to gastric disease progression. Even if these oral microbes do not directly translocate to or persist within the gastric mucosa, they may exert indirect effects, for example, by promoting chronic inflammation or modulating mucosal immune responses, thereby contributing to disease-associated gastric alterations. These findings support the potential of the oral microbiota as a noninvasive biomarker source for risk stratification or disease staging, particularly for reflecting disease stage rather than serving as a direct surrogate for the gastric microbiome.

However, the findings of this study should be interpreted in light of several methodological considerations. First, formal dental examinations were not performed, and detailed information on participants’ dental and oral health status, including the presence of periodontal disease, overall dental condition, and oral hygiene habits, was not available. Although buccal swab samples primarily reflect mucosal-associated oral microbiota rather than dental plaque-associated communities, oral health-related factors may still have influenced oral microbial profiles. In addition, although patients were instructed to discontinue antibiotics and proton pump inhibitors prior to endoscopy, detailed verification of over-the-counter antacid use was not systematically performed. Therefore, residual confounding related to oral health and medication exposure cannot be completely excluded when interpreting microbiome-related findings. Furthermore, this study employed a cross-sectional design, which precludes causal inference regarding the observed associations between microbial features and disease stage. Consequently, it remains unclear whether the identified microbial alterations contribute to disease progression or arise as a consequence of gastric pathological changes.

## 5. Conclusions

In this study, we compared the oral and gastric microbiomes of patients across five gastric cancer-related histological stages. Pairwise differential abundance analyses identified several potential microbial markers, beyond *Helicobacter*, that could discriminate between disease stages. Notably, taxa typically associated with the intestinal microbiota were enriched in the antrum and body of cancerous samples in the integrated analysis. However, when *H. pylori*-negative cases were analyzed separately, intestine-associated taxa such as *Faecalibacterium* were less abundant in advanced gastric cancer, suggesting distinct microbial trajectories during carcinogenesis in the absence of *H. pylori*. In addition, several oral microbial taxa emerged as potential markers, highlighting the possibility of using oral microbiota as non-invasive indicators of gastric carcinogenesis stages.

Despite these findings, the identified taxa should be regarded as exploratory biomarkers rather than candidates for immediate clinical application. Given that the human microbiota is influenced by multiple factors, including geographic location, ethnicity, sex, dietary habits, and host genetics, the generalizability and applicability of these markers require careful validation. Future studies should focus on independent validation in diverse populations and on integrating microbiome data with relevant clinical parameters, such as dental status, inflammatory markers, and other clinical metadata. Such efforts will be essential to determine whether these microbial signatures can be reliably translated into clinically meaningful tools for gastric cancer risk assessment and stage-specific diagnosis.

## Data Availability

The data presented in this study are openly available in [the National Center for Biotechnology Information (NCBI) Sequence Read Archive (SRA)] at [https://dataview.ncbi.nlm.nih.gov/object/PRJNA1380972?reviewer=6r16hgh7st9emutg10bcrnmu8v (accessed on 14 December 2025)], reference number [PRJNA1380972].

## Supplementary Materials

The following supporting information can be downloaded at https://www.mdpi.com/article/10.3390/microorganisms14010209/s1. Figure S1. Boxplots representing the Firmicutes/Bacteroidetes (F/B) ratio in each histological stage from (A) antrum, (B) body, and (C) oral samples. Letters above the boxes indicate the results of Tukey’s post hoc test. Figure S2. Boxplots representing the number of OTUs (operational taxonomic units) in each histological stage from (A) antrum, (B) body, and (C) oral samples. Letters above the boxes indicate the results of Tukey’s post hoc test. Figure S3. Boxplots representing the Shannon diversity in each histological stage from (A) antrum, (B) body, and (C) oral samples. Letters above the boxes indicate the results of Tukey’s post hoc test. Table S1. Overview of participant information, including demographic, clinical, and sample metadata (provided in a separate Excel file). Table S2. PERMANOVA (ADONIS) and pairwise PERMANOVA results showing the differences in microbial community structure of samples collected from different points. Table S3. Results of the envfit analysis showing the correlations between personal characteristics and the nMDS ordination (Figure 1A). HP: *Helicobacter pylori* status (presence vs. absence). Table S4. PERMANOVA (ADONIS) and pairwise PERMANOVA results showing the differences in microbial community structure of antrum samples at different disease stages. Table S5. Results of the envfit analysis showing the correlations between personal characteristics and the nMDS ordination of antrum samples (Figure 1B). HP: *Helicobacter pylori* status (presence vs. absence). Table S6. PERMANOVA (ADONIS) and pairwise PERMANOVA results showing the differences in microbial community structure of body samples at different disease stages. Table S7. Results of the envfit analysis showing the correlations between personal characteristics and the nMDS ordination of body samples (Figure 1C). HP: *Helicobacter pylori* status (presence vs. absence). Table S8. PERMANOVA (ADONIS) and pairwise PERMANOVA results showing the differences in microbial community structure of oral samples at different disease stages. Table S9. Results of the envfit analysis showing the correlations between the personal characteristics and the nMDS ordination of oral samples (Figure 1D). HP: *Helicobacter pylori* status (presence vs. absence). Table S10. PERMANOVA (ADONIS) and pairwise PERMANOVA results for *Helicobacter pylori*-negative patients, showing the differences in microbial community structure across sampling sites. Table S11. Results of envfit analysis in *Helicobacter pylori*-negative patients, illustrating the correlations between personal characteristics and nMDS ordination (Figure 2A). Table S12. PERMANOVA (ADONIS) and pairwise PERMANOVA results for the antrum samples from *Helicobacter pylori*-negative patients across different histological stages. Table S13. Envfit analysis results for the antrum samples from *Helicobacter pylori*-negative patients, showing correlations between personal characteristics and nMDS ordination (Figure 2B). Table S14. PERMANOVA (ADONIS) and pairwise PERMANOVA results for the body samples from *Helicobacter pylori*-negative patients across different histological stages. Table S15. Envfit analysis results for the body samples from *Helicobacter pylori*-negative patients, showing correlations between personal characteristics and nMDS ordination (Figure 2C). Table S16. PERMANOVA (ADONIS) and pairwise PERMANOVA results for the oral samples from *Helicobacter pylori*-negative patients across different histological stages. Table S17. Envfit analysis results for the oral samples from *Helicobacter pylori*-negative patients, showing correlations between personal characteristics and nMDS ordination (Figure 2D).

## Abbreviations

The following abbreviations are used in this manuscript:

GC	Gastric cancer
IM	Intestinal metaplasia
LGD	Low-grade dysplasia
HGD	High-grade dysplasia
EGC	Early gastric cancer
AGC	Advanced gastric cancer
NCBI	National Center for Biotechnology Information
SRA	Sequence Read Archive
OTU	Operational taxonomic units
ANOVA	Analysis of variance
HSD	Honestly significant difference
nMDS	Non-metric multidimensional scaling
PERMANOVA	Permutational multivariate analysis of variance
SCFA	Short-chain fatty acid
