# Supplementary material for: Characterization of the Oral and Stomach Microbial Community Structure in Patients with Intestinal Metaplasia, Dysplasia, and Gastric Cancer Through High-Throughput Sequencing
Source: Microorganisms. 2026 Jan 16;14(1):209. doi: 10.3390/microorganisms14010209 (PMC12844037; doi:10.3390/microorganisms14010209)
Supplement: Supplementary file 1 [file microorganisms-14-00209-s001.zip › Supplementary Materials.pdf]

Figure S1. Boxplots representing the Firmicutes/Bacteroidetes (F/B) ratio in each histological stage from (A) antrum, (B) body, and (C) oral samples. Letters above the boxes indicate the results of Tukey's post-hoc test.

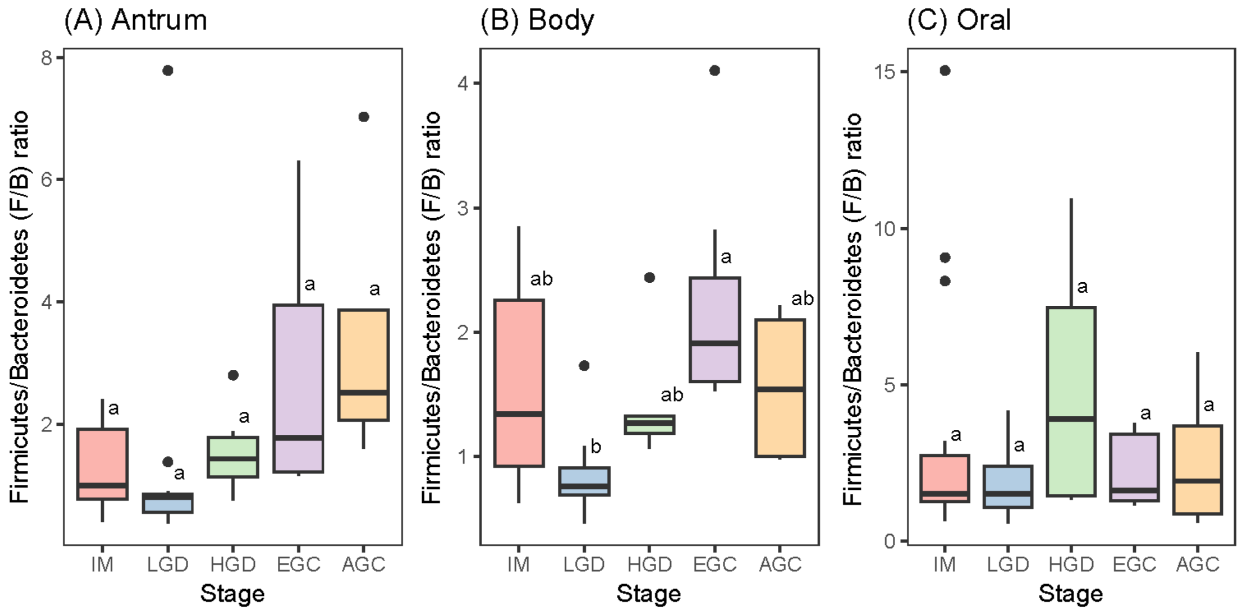

Figure S2. Boxplots representing the number of OTUs (operational taxonomic units) in each histological stage from (A) antrum, (B) body, and (C) oral samples. Letters above the boxes indicate the results of Tukey's post-hoc test.

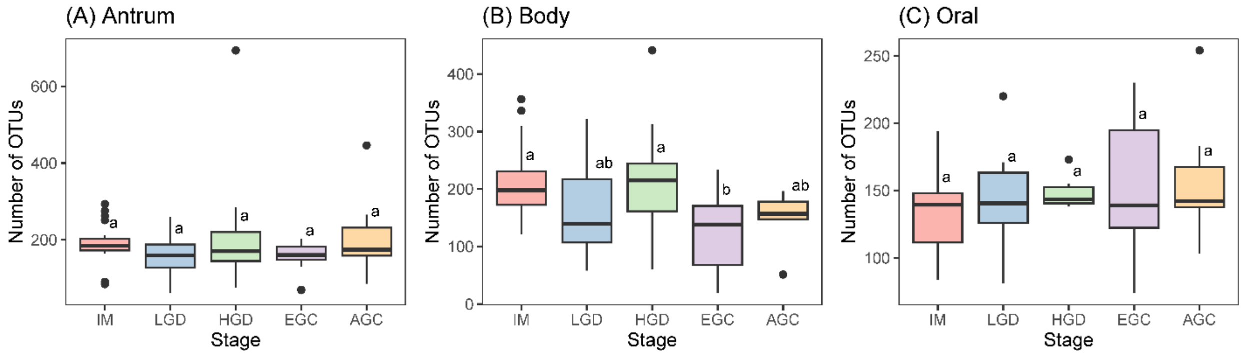

Figure S3. Boxplots representing the Shannon diversity in each histological stage from (A) antrum, (B) body, and (C) oral samples. Letters above the boxes indicate the results of Tukey’s post-hoc test.

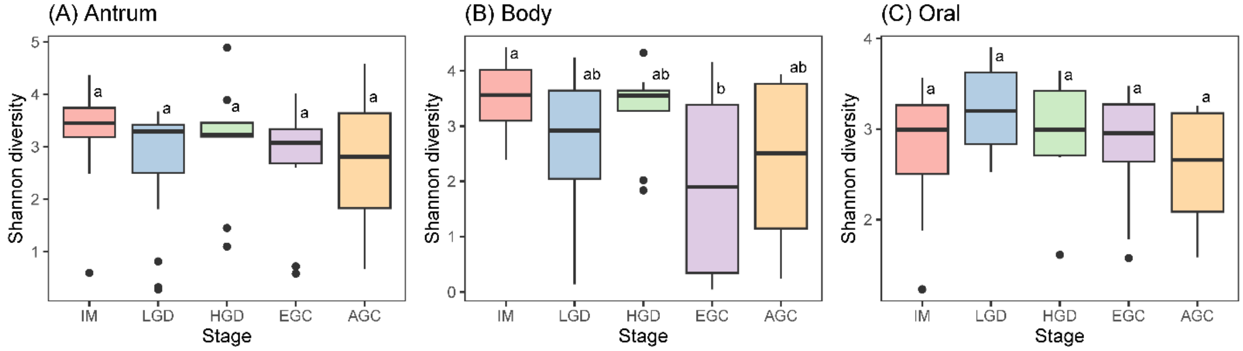

Table S1. Overview of participant information, including demographic, clinical, and sample metadata (provided in a separate Excel file).

Table S2. PERMANOVA (ADONIS) and pairwise PERMANOVA results showing the differences in microbial community structure of samples collected from different points.

Global test result	R^2= 0.114, p-value = 0.001	
Pairwise test result		
Pair	R^2	Adjusted p-value
Antrum vs Body	0.014	0.078
Antrum vs Oral	0.112	0.003
Body vs Oral	0.141	0.003

Table S3. Results of the envfit analysis showing the correlations between personal characteristics and the nMDS ordination (Fig. 1A). HP: *Helicobacter pylori* status (presence vs absence).

	NMDS1	NMDS2	R^2	p-value
HP	0.627	0.779	0.237	0.001
Sex	-0.371	-0.928	0.052	0.008
Age	-0.958	-0.288	0.007	0.524
Drink	0.076	-0.997	0.022	0.123
Smoke	-0.535	-0.845	0.010	0.364
BMI	-0.826	-0.563	0.027	0.072

Table S4. PERMANOVA (ADONIS) and pairwise PERMANOVA results showing the differences in microbial community structure of antrum samples at different diseases stages.

Global test result	R^2= 0.076, p-value = 0.044	
Pairwise test result		
Pair	R^2	Adjusted p-value
LGD vs HGD	0.054	1
LGD vs EGC	0.046	1
LGD vs IM	0.048	0.15
LGD vs AGC	0.062	0.72
HGD vs EGC	0.055	1
HGD vs IM	0.031	1
HGD vs AGC	0.064	1
EGC vs IM	0.039	1
EGC vs AGC	0.037	1
IM vs AGC	0.048	1

Table S5. Results of the envfit analysis showing the correlations between personal characteristics and the nMDS ordination of antrum samples (Fig. 1B). HP: *Helicobacter pylori* status (presence vs absence).

	NMDS1	NMDS2	R^2	p-value
HP	-0.242	0.970	0.384	0.001
Sex	0.259	-0.966	0.053	0.181
Age	0.436	-0.900	0.007	0.821
Drink	0.940	-0.340	0.017	0.585
Smoke	-0.802	-0.598	0.022	0.493
BMI	-0.478	-0.878	0.050	0.212

Table S6. PERMANOVA (ADONIS) and pairwise PERMANOVA results showing the differences in microbial community structure of body samples at different diseases stages.

Global test result	R^2= 0.099, p-value = 0.008	
Pairwise test result		
Pair	R^2	Adjusted p-value
LGD vs HGD	0.053	1
LGD vs EGC	0.033	1
LGD vs IM	0.057	0.25
LGD vs AGC	0.033	1
HGD vs EGC	0.101	0.24
HGD vs IM	0.032	1
HGD vs AGC	0.082	1
EGC vs IM	0.104	0.01
EGC vs AGC	0.032	1
IM vs AGC	0.055	0.75

Table S7. Results of the envfit analysis showing the correlations between personal characteristics and the nMDS ordination of body samples (Fig. 1C). HP: *Helicobacter pylori* status (presence vs absence).

	NMDS1	NMDS2	R^2	p-value
HP	0.919	0.394	0.582	0.001
Sex	-0.951	-0.308	0.113	0.030
Age	-0.629	-0.778	0.049	0.209
Drink	-0.934	0.357	0.042	0.290
Smoke	-0.282	-0.959	0.028	0.412
BMI	-0.303	-0.953	0.027	0.415

Table S8. PERMANOVA (ADONIS) and pairwise PERMANOVA results showing the differences in microbial community structure of oral samples at different diseases stages.

Global test result	R^2= 0.078, p-value = 0.012	
Pairwise test result		
Pair	R^2	Adjusted p-value
LGD vs HGD	0.043	1
LGD vs EGC	0.041	0.88
LGD vs IM	0.064	0.01
LGD vs AGC	0.073	0.07
HGD vs EGC	0.031	1
HGD vs IM	0.033	1
HGD vs AGC	0.053	1
EGC vs IM	0.037	1
EGC vs AGC	0.038	1
IM vs AGC	0.046	1

Table S9. Results of the envfit analysis showing the correlations between the personal characteristics and the nMDS ordination of oral samples (Fig. 1D). HP: *Helicobacter pylori* status (presence vs absence).

	NMDS1	NMDS2	R^2	p-value
HP	-0.979	0.204	0.121	0.022
Sex	0.825	0.566	0.084	0.060
Age	0.124	-0.992	0.055	0.153
Drink	0.851	0.525	0.055	0.171
Smoke	0.911	0.413	0.058	0.149
BMI	0.011	1.000	0.072	0.081

Table S10. PERMANOVA (ADONIS) and pairwise PERMANOVA results for *Helicobacter pylori*–negative patients, showing the differences in microbial community structure across sampling sites.

Global test result	R^2= 0.108, p-value = 0.001	
Pairwise test result		
Pair	R^2	Adjusted p-value
Antrum vs Body	0.010	1
Antrum vs Oral	0.110	0.003
Body vs Oral	0.133	0.003

Table S11. Results of envfit analysis in *Helicobacter pylori*–negative patients, illustrating the correlations between personal characteristics and nMDS ordination (Fig. 2A).

	NMDS1	NMDS2	R^2	p-value
Sex	0.117	-0.993	0.011	0.513
Age	-0.831	-0.556	0.028	0.131
Drink	0.911	-0.413	0.007	0.617
Smoke	-0.181	0.984	0.029	0.151
BMI	-0.779	0.627	0.014	0.389

Table S12. PERMANOVA (ADONIS) and pairwise PERMANOVA results for the antrum samples from *Helicobacter pylori*-negative patients across different histological stages.

Global test result	R^2= 0.104, p-value = 0.088	
Pairwise test result		
Pair	R^2	Adjusted p-value
LGD vs HGD	0.107	0.11
LGD vs EGC	0.110	0.14
LGD vs IM	0.050	0.24
LGD vs AGC	0.106	0.62
HGD vs EGC	0.119	0.72
HGD vs IM	0.032	1
HGD vs AGC	0.107	1
EGC vs IM	0.042	1
EGC vs AGC	0.099	1
IM vs AGC	0.035	1

Table S13. Envfit analysis results for the antrum samples from *Helicobacter pylori*–negative patients, showing correlations between personal characteristics and nMDS ordination (Fig. 2B).

	NMDS1	NMDS2	R^2	p-value
Sex	-0.424	0.906	0.044	0.395
Age	0.975	-0.222	0.040	0.457
Drink	-0.507	0.862	0.150	0.028
Smoke	-0.755	0.655	0.120	0.072
BMI	-0.001	1.000	0.038	0.464

Table S14. PERMANOVA (ADONIS) and pairwise PERMANOVA results for the body samples from *Helicobacter pylori*-negative patients across different histological stages.

Global test result	R^2= 0.095, p-value = 0.353	
Pairwise test result		
Pair	R^2	Adjusted p-value
LGD vs HGD	0.096	0.95
LGD vs EGC	0.082	1
LGD vs IM	0.050	1
LGD vs AGC	0.093	1
HGD vs EGC	0.105	1
HGD vs IM	0.038	1
HGD vs AGC	0.127	1
EGC vs IM	0.030	1
EGC vs AGC	0.101	1
IM vs AGC	0.034	1

Table S15. Envfit analysis results for the body samples from *Helicobacter pylori*–negative patients, showing correlations between personal characteristics and nMDS ordination (Fig. 2C).

	NMDS1	NMDS2	R^2	p-value
Sex	0.414	-0.910	0.058	0.305
Age	0.990	-0.141	0.145	0.044
Drink	-0.401	-0.916	0.032	0.519
Smoke	0.572	0.820	0.045	0.425
BMI	0.990	0.144	0.033	0.521

Table S16. PERMANOVA (ADONIS) and pairwise PERMANOVA results for the oral samples from *Helicobacter pylori*-negative patients across different histological stages.

Global test result	R^2= 0.103, p-value = 0.052	
Pairwise test result		
Pair	R^2	Adjusted p-value
LGD vs HGD	0.073	1
LGD vs EGC	0.067	1
LGD vs IM	0.062	0.03
LGD vs AGC	0.086	1
HGD vs EGC	0.075	1
HGD vs IM	0.028	1
HGD vs AGC	0.136	1
EGC vs IM	0.047	1
EGC vs AGC	0.087	1
IM vs AGC	0.051	1

Table S17. Envfit analysis results for the oral samples from *Helicobacter pylori*–negative patients, showing correlations between personal characteristics and nMDS ordination (Fig. 2D).

	NMDS1	NMDS2	R^2	p-value
Sex	0.622	-0.783	0.104	0.088
Age	0.965	0.263	0.015	0.749
Drink	0.808	-0.589	0.040	0.394
Smoke	0.984	-0.178	0.098	0.092
BMI	0.012	-1.000	0.064	0.223
